# Diuretic responses to Ringer's solution and 20% albumin at different arterial pressures

**DOI:** 10.14814/phy2.70069

**Published:** 2024-10-07

**Authors:** Robert G. Hahn, Markus Zdolsek, Michaela Gunnström, Emma Hasselgren, Joachim H. Zdolsek

**Affiliations:** ^1^ Karolinska Institute at Danderyds Hospital (KIDS) Stockholm Sweden; ^2^ Department of Biomedical and Clinical Sciences (BKV) Linköping University Linköping Sweden; ^3^ Perioperative Medicine and Intensive Care Karolinska University Hospital Solna Sweden

**Keywords:** blood, fluid, fluid balance, hemodilution, hyper‐oncotic albumin, infusion, pharmacokinetics, Ringer

## Abstract

Intravenous volume loading is a common treatment when hypovolemia is a potential cause of oliguria. We studied whether the effectiveness of Ringer's solution and 20% albumin in inducing diuresis differs depending on the mean arterial pressure (MAP). For this purpose, volume kinetic analysis was performed based on urine output and hemoglobin‐derived plasma dilution obtained during and after 136 infusions of Ringer and 85 infusions of 20% albumin. Covariance analysis quantified the diuretic response at different arterial pressures. The results show that the diuretic response to a known plasma volume expansion was greater for Ringer's solution above a MAP of 70 mmHg, while 20% albumin was significantly more effective at lower pressures (*p* < 0.03). Simulations of the urinary output in response to infusion of a predefined fluid volume yielded superior efficacy for 20% albumin when the MAP was low, while Ringer's was similarly effective when the MAP averaged 100 mmHg. In conclusion, urine output in response to plasma volume expansion with 20% albumin was similar to, or even stronger, than that of Ringer's solution when the MAP was below 70 mmHg.

## Graphical abstract

Intravenous fluid is a common treatment in oliguric patients who may be hypovolemic. The diuretic responses to Ringer's and 20% albumin were compared in 221 subjects. The diuretic response was greater for Ringer's down to MAP of 70 mmHg. The diuretic response was greater for 20% albumin when MAP was below 70 mmHg. 20% albumin was more effective in creating prolonged diuretic effects (>1 h).

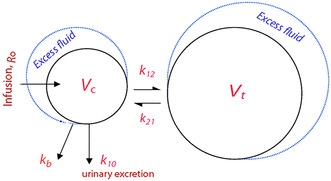

## 1 INTRODUCTION

Oliguria is a common sign in perioperative and intensive care that prompts action from the medical team. Oliguria has a multifactorial background and is associated with a minor but statistically significant increase in the risk of acute kidney injury (AKI) (Tallarico et al., 2024). Low urine flow is an appropriate physiological response in patients with low intravascular volume; therefore, intravenous fluid can be used to induce diuresis when the clinical scenario is compatible with hypovolemia (Zarbock et al., 2018). Pretesting of fluid responsiveness is recommended (passive leg raising test) because fluid treatment is not always helpful (Inkinen et al., 2022; Legrand et al., 2016).

The effectiveness of a fluid bolus in inducing diuresis depends not only on the clinical and hemodynamic situation, but also on the type of fluid. The choice of fluid is particularly difficult in hypotensive patients with oliguria and peripheral edema. Crystalloids are the standard solutions used for this purpose, while colloids are more controversial (Inkinen et al., 2022). Hyper‐oncotic albumin might be useful in this setting because it counteracts peripheral edema by redistributing interstitial volume to the plasma without greatly increasing the fluid balance (Zdolsek & Hahn, 2022). General anesthesia increases the likelihood of oliguria by decreasing the arterial pressure (Hahn, 2017) and further promotes peripheral edema by a depressive effect on the lymphatic pumping (Dull et al., 2024). Therefore, the choice between crystalloid fluid and hyper‐oncotic albumin is important when using PV expansion to treat oliguria in the perioperative period.

The objective of this study was to compare the diuretic response to the plasma volume (PV) expansion induced by Ringer's solution (large‐volume fluid administration) with that of 20% albumin (small‐volume fluid administration) depending on the mean arterial pressure (MAP). The hypothesis was that differences in diuretic response between Ringer's solution and 20% albumin vary with the MAP. The justification of this hypothesis is that previous work shows that the urine output declines sharply when MAP is low (Hahn, 2017) while this covariance has been difficult to demonstrate for 20% albumin (Hasselgren et al., 2019; Zdolsek et al., 2022). Thus, it is possible that 20% albumin is more effective than crystalloid fluid in inducing diuresis in hypotensive states.

## 2 MATERIALS AND METHODS

### 2.1 Study overview

The study material was derived from a database of intravenous infusion experiments in humans in which the purpose was always to study fluid volume kinetics. The subjects had not undergone hemodynamic manipulation (or had only minimal manipulation) and were free from physiological alterations other than, at times, general anesthesia and mild inflammation. The exclusion criteria were age <18 years, poor health status (American Society of Anesthesiologists (ASA) Classes III–IV), severe allergies, impaired kidney function, and heart failure. The first author planned and supervised the studies and confirm that standardized methods of providing fluid and collecting data were followed. All experiments were performed in accordance with the Declaration of Helsinki and each subject provided written informed consent to participate before any experiment was initiated.

The study included 136 experiments with Ringer's acetate/lactate solution (Drobin & Hahn, 1999, 2002; Ewaldsson & Hahn, 2005; Hahn et al., 2011; Hahn & Olsson, 2022; Li et al., 2012; Svensén et al., 1999; Zdolsek et al., 2012) and 85 infusions of 20% albumin (Gunnström et al., 2022; Hasselgren et al., 2019; Zdolsek et al., 2020, 2022). Two‐thirds of the experiments were performed in healthy volunteers while the other infusions were performed in clinical patients when undergoing elective surgery under general anesthesia. Bleeding was minor (50–150 mL).

Complete studies were included, but only the control experiments were used if the protocol included deliberate dehydration or withdrawal of blood (in addition to sampling volume). No diuretic drugs were used, and only one infusion fluid was given during each experiment. All subjects were considered euvolemic when the infusions were initiated. Reporting adhered to the STROBE checklist.

### 2.2 Procedures

The volunteers were allowed to ingest one glass of liquid on the morning of the experiment, which usually started between 8 and 9 a.m. to compensate for overnight evaporation. Surgical patients were allowed to ingest liquid up to 2 h before the operation and received no premedication or diazepam by mouth. Data obtained before or during anesthesia induction and after awakening were not included. No fluid was given during the induction.

All experiments were performed in a similar manner. The principle was that 1.5–2.0 L of acetated Ringer's solution (electrolyte content in mmol/L: Na^+^ 130, Cl^−^ 110, K^+^ 4, Ca^2+^ 2, Mg^+^ 1, and acetate^−^ 30) or 3 mL/kg of 20% albumin (approximately 200 mL, Albunorm, Octopharma, Na^+^ 144–160 mmol/L) over 30 min was infused intravenously in proportion to body weight. See Table 1 for details.

**TABLE 1:** Demographic and basic experimental data. Mean (SD) or the incidence is shown.

	Ringer's	20% albumin	*P*
Basic data			
Experiments (*N*)	136	84	
Males (%)	75 (56%)	39 (46%)	0.29
Age (years)	37 (13)	41 (18)	0.03
Body weight (kg)	72.9 (13.2)	77.6 (15.9)	0.02
Length (cm)	175 (10)	174 (11)	0.45
Body mass index (kg/m^2^)	25.2 (3.3)	25.6 (4.4)	0.43
During anesthesia (%)	54 (41%)	15 (18%)	0.001
Infused volume (mL)	1676 (447)	232 (48)	0.001
Infusion rate (mL/min)	64 (32)	7.0 (2.6)	0.001
Length of experiment (min)	148 (58)	314 (32)	–
Data points in kinetic analysis (N)	3198	1256	–
Data points/experiment (mean)	24	15	–
Urine portions measured (N)	395	206	–
Urine portions/experiment (mean)	3	2.5	–
Measurements			
Blood Hb at baseline (g/L)	128 (14)	124 (18)	0.06
MAP when starting infusion (mmHg)	80 (13)	84 (13)	0.02
MAP during experiment (mmHg)^a^	80 (12)	84 (13)	0.02
Awake (mL)	86 (10)	87 (12)	0.25
Anesthetized (mL)	72 (11)	72 (12)	0.88
Patients with urine flow **≤**0.5 mL/min	29 (21%)	10 (12%)	0.08
Urine output (mL)	607 (494)	613 (354)	0.92
Awake (mL)	945 (390)	597 (352)	0.001
Anesthetized (mL)	65 (13)	678 (380)	0.001
Urine portions with flow **≤**0.5 mL/min (N)	90 (22%)	15 (7%)	0.001
Urine portions with flow **≤**0.3 mL/min (N)	43 (11%)	4 (2%)	0.002
MAP when urine flow **≤**0.5 mL/min (mmHg)	76 (13)	74 (11)	0.74
MAP when urine flow ≥0.5 mL/min (mmHg)	83 (15)	84 (14)	0.45

*Note*: Statistics were performed with one‐way ANOVA or the chi‐square test, where appropriate.

Abbreviation: N, number.

^a^ Based on the mean for each experiment.

Hemoglobin (Hb) measurements analyzed 2–3 mL of venous blood obtained from a cannula in the cubital vein on 15–30 occasions over 2–6 h. Specifically, blood was sampled every 5 min during each infusion and for 30 min after. Thereafter, the interval was increased to 10–30 min. Hb was measured at the hospital's certified clinical chemistry laboratory with a coefficient of variation (CV) of approximately 1%. When 20% albumin was infused, plasma albumin and urinary creatinine were measured using a Cobas® 8000 (Roche Diagnostics, Basel, Switzerland) with CVs of 3.2% and 1.9%, respectively.

The MAP and heart rate were measured at the time of each blood sampling using an invasive or noninvasive automatic hemodynamic monitor. The urine output was measured 2–3 times per experiment.

### 2.3 Diuretic response

How to express the diuretic response to fluid‐induced PV expansion is not clearly defined in the literature. In the present study, this diuretic response is expressed as (1) the total measured urine output at the end of the experiment, (2) as the kinetic parameter *k*_10_, and (3) as the urine output divided by the infused fluid volume with and without being modeled to achieve a predetermined PV expansion. The two latter approaches require a kinetic analysis of the distribution of the infused fluid in the whole body.

Urinary excretion is the chief mechanism by which the body corrects expansion of the blood volume above baseline. The parameter *k*_10_ is claimed to be a more sophisticated measure of diuretic response than the crude urine output alone because it relates the measured urine flow directly to the fluid‐induced PV expansion occurring during the same period of time. This parameter is free from several confounders that may affect the measured urine output, such as variations in infused fluid volume (fluid is given in proportion to body weight), infusion time, and follow‐up time. As *k*_10_ considers the PV expansion per se, it is free from the influence of variable transcapillary leakage, recruitment of fluid from the interstitium (primarily lymph), and elimination half‐life. The parameter was calculated similarly with the Ringer and 20% albumin experiments and, technically, *k*_10_ yields the urine flow in mL/min if multiplied by the PV expansion at any time. Once *k*_10_ is obtained, the expected urine output in response to a predetermined PV expansion can be simulated. How the kinetic analysis was carried out is described in the next paragraph.

### 2.4 Kinetic analyses

Kinetic models were constructed to mimic human physiology (Guyton & Hall, 1996). A diagram of the volume kinetics of the Ringer solution is shown in Figure S1a in File S1. Infused fluid is distributed between two body fluid spaces of which the central space (*V*_c_) represents the plasma. Rate constants govern the flow rate to and from the central and the peripheral space, which occurs in direct proportion the volume expansion of the respective space. Elimination takes place from the plasma through urinary excretion, which rate is governed by a rate constant denoted *k*_10_ (Hahn, 2020).

Figure S1B shows diagram of the kinetic model used to analyze the fluid shifts when infusing 20% albumin. Here, extravascular fluid is absorbed to the plasma due to the hyper‐oncotic nature of 20% albumin. Fluid leaks back into the extravascular space in proportion to the modeled PV expansion. Infused fluid is eliminated by urinary excretion at the rate constant *k*_10_, which is then obtained in the same way as with Ringer's kinetics. The build‐up and operation of these models are discussed in detail in previous studies (Hahn, 2020; Hahn et al., 2019).

The fractional plasma dilution, based on the changes in Hb, was used to indicate the central volume expansion resulting from the infusions. This calculation was corrected for the losses of Hb with blood sampling and surgery (Ewaldsson & Hahn, 2005). The procedures used for these calculations and the differential equations for the kinetic models are given in File S1.

### 2.5 Calculations

The kinetic model was fitted to all measurements of plasma dilution and urinary output using Phoenix software version 8.3.4 for nonlinear mixed effects (Pharsight, St. Louis, MO). One analysis was made for Ringer's solution and another for 20% albumin. “First‐order conditional estimation extended least squares” was used as the search routine. The criterion for accepting a covariate was that its inclusion reduced the −2 LL (log likelihood) for the model by >6.6 points (*p* < 0.01) (Owen & Fiedler‐Kelly, 2014).

The value of *k*_10_ and the other model parameters (Figure S1) resulted in different values for each subject depending on the influences of individual‐specific covariates, which included the MAP for both fluids but also other measured variables. The principles for the covariate search are explained in detail in File S1. The reported MAP and *k*_10_ are the time‐corrected mean values based on all measurements of MAP, urine output, and the volume expansion *V*_c_ for each experiment, as given by the Phoenix software. As mentioned, *k*_10_ is yielded as the urine divided by the volume expansion of *V*_c_, the latter of which represents the plasma volume. The mathematical changes of the kinetic parameters that are due to covariates are also explained in File S1. Simulations were performed using MATLAB R2020a (Math Works, Natick, MA, USA).

The data are reported as the means (standard deviations), and the two groups were compared with one‐way analysis of variance. Categorical data were evaluated using a chi‐squared test. The relationships between the continuous variables were studied by linear regression. *p* < 0.05 was considered statistically significant.

Kinetic parameters were reported via the best estimate and the 95% confidence interval (CI). These data and the significance levels for the inclusion of covariates were taken from the Phoenix software.

## 3 RESULTS

### 3.1 Incidence of low urine flow

Basic data are summarized in Table 1, and the original data are given in Files S2 and S3. The urine flow varied between 0.1 and 10 mL/min (median, 1.7). Low flow (≤0.5 mL/min) was found in 17% of the 601 urine collections. All these events occurred during the perioperative period. Oliguria (≤0.3 mL/min) was found in 8% of the collections.

The MAP was lower during periods of low urine flow than during other time periods, both for Ringer (*p* < 0.001) and 20% albumin (*p* < 0.01; Table 1).

**FIGURE 1:**
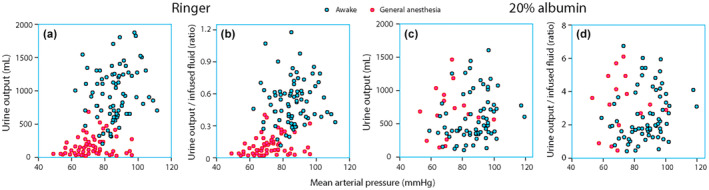
Urine output versus mean arterial pressure (MAP). (a) Relationship between the urine output measured at the end of each experiment with Ringer's solution and the average MAP during the entire experiment as calculated by the Phoenix program. (b) The urine output versus the amount of infused fluid. (c) Same plot as (a) but for 20% albumin. (d) Same plot as (b) but for 20% albumin. Note the ability of 20% albumin to induce diuresis that far exceeds the infused volume. Each point represents one experiment.

Figure 1 shows the relationship between the total urine output and MAP during all individual infusion experiments with Ringer and 20% albumin.

### 3.2 Kinetic analysis

**FIGURE 2:**
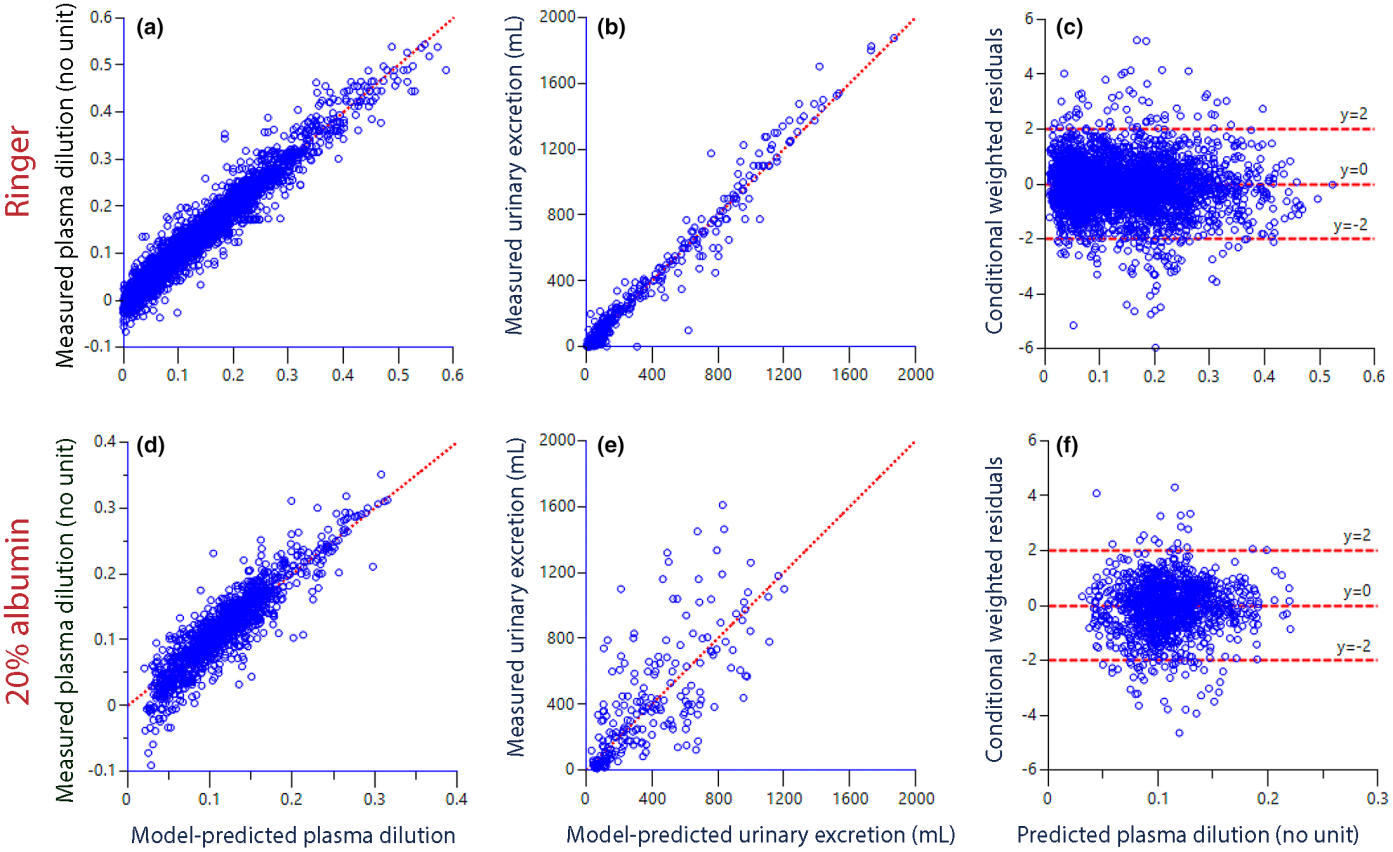
Goodness‐of‐fit, that is, the ability of the kinetic model to recreate the input data. These data are for the Ringer's infusions (*top row*) and 20% albumin (lower row). (a) The relationship between the measured plasma dilution and the model‐predicted plasma dilution when all covariates were considered. The line of unity is shown in red. (b) Same plot for the urinary excretion. (c) The conditional weighted residuals (CWRES) for the plasma dilution as predicted by the model without covariates considered. The irregular red lines show ±2 SD for the residuals. (d–f) Same plots but for 20% albumin. Each point is one measurement.

Kinetic analyses were performed to refine the quantification of urine output by calculating a parameter *k*_10_, which is the ratio between the measured urine output and the PV expansion over time. This strengthened the correlation between MAP and urine output by considering the overall distribution of infused model. Two kinetic models were used for this purpose, one for Ringer and another for 20% albumin; they are illustrated in Figure S1. These models were successfully fitted to all infusion experiments in a single run for each fluid. The final parameter estimates are given in Tables S1 and S2 and their ability to recreate the input data (“goodness of fit”) is illustrated in Figure 2.

**FIGURE 3:**
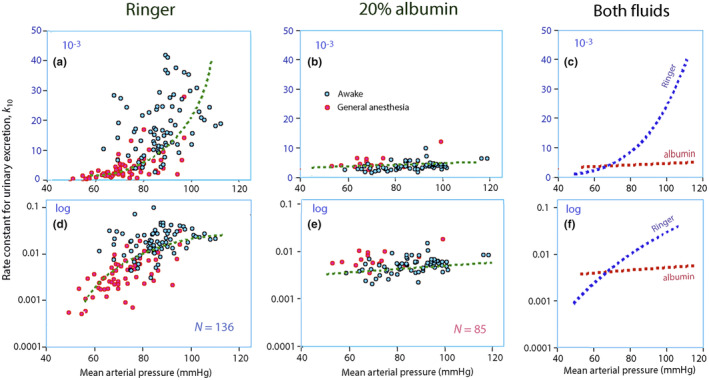
Arterial pressure versus *k*_10_. The mean arterial pressure (MAP) versus the covariance‐adjusted rate constant for urinary excretion (*k*_10_) for (a) Ringer's solution and (b) 20% albumin. (c) Superposition of the regression lines for the two fluids. (d–f) Same plots but on the logarithmic scale. Each point is one experiment.

The covariate analysis confirmed that a low MAP promoted a poorer diuretic response (lower *k*_10_) for both the Ringer and albumin infusions, but the slope for the correlation between MAP and *k*_10_ was less steep for albumin (Figure 3a–c).

Logarithmic transformation clarified that *k*_10_ was greater for Ringer's than for albumin down to a MAP of approximately 70 mmHg (Figure 3d–f). Specifically, *k*_10_ was significantly greater for 20% albumin when the MAP was ≤68.0 mmHg (median 3.7 × 10^−3^ min^−1^ versus 2.2 × 10^−3^ for Ringer's; 38 experiments; *p* < 0.03).

**FIGURE 4:**
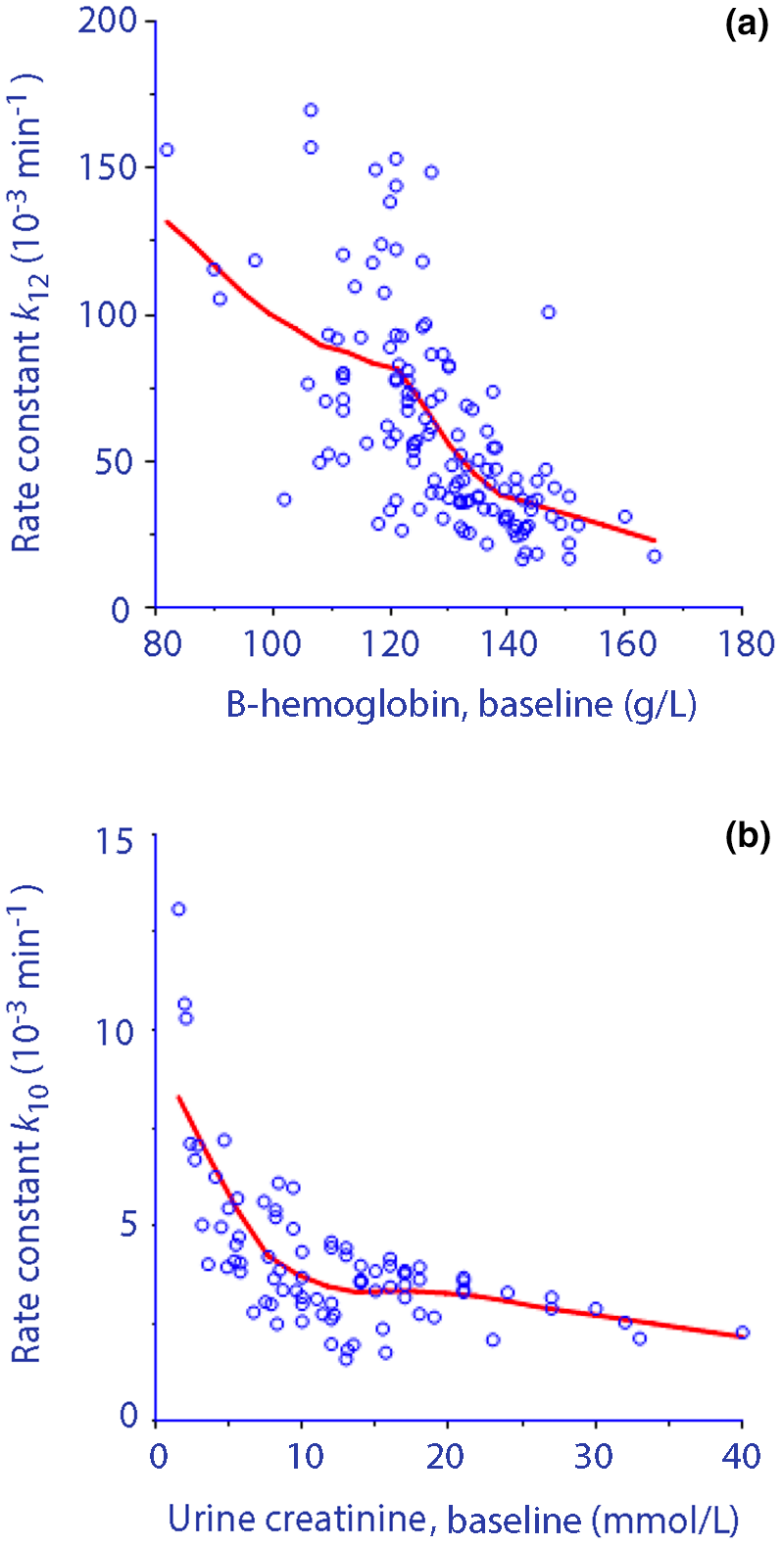
Strong covariates. (a) Relationship between the blood hemoglobin (Hb) concentration measured just before the Ringer infusions and the rate constant for distribution of fluid (*k*_12_). The reason for this covariance is probably that a high Hb concentration increases the blood viscosity. (b) Relationship between the urine creatinine concentration and the covariate‐corrected rate constant for urinary excretion (*k*_10_) when 20% albumin was infused. Red lines show the logically weighted scatterplot smoothing line.

Other covariates included that a high Hb concentration was followed by a slower distribution of Ringer's solution (lower *k*_12_; Figure 4a) and that a high urinary creatinine concentration decreased the urinary flow when 20% albumin was used (Figure 4b). In each subject, the influence of these covariances on *k*_10_ was corrected by the kinetic model.

Most of the lowest values for MAP and *k*_10_ were obtained during general anesthesia, which Figure 3d suggests that the relationship between these two parameters represent a relatively stable continuum in the awake and anesthetized states.

Table 1 shows that general anesthesia was associated with a lower MAP in both groups, while the urine output was lower only when Ringer's solution was infused.

### 3.3 Simulating diuretic responses

**FIGURE 5:**
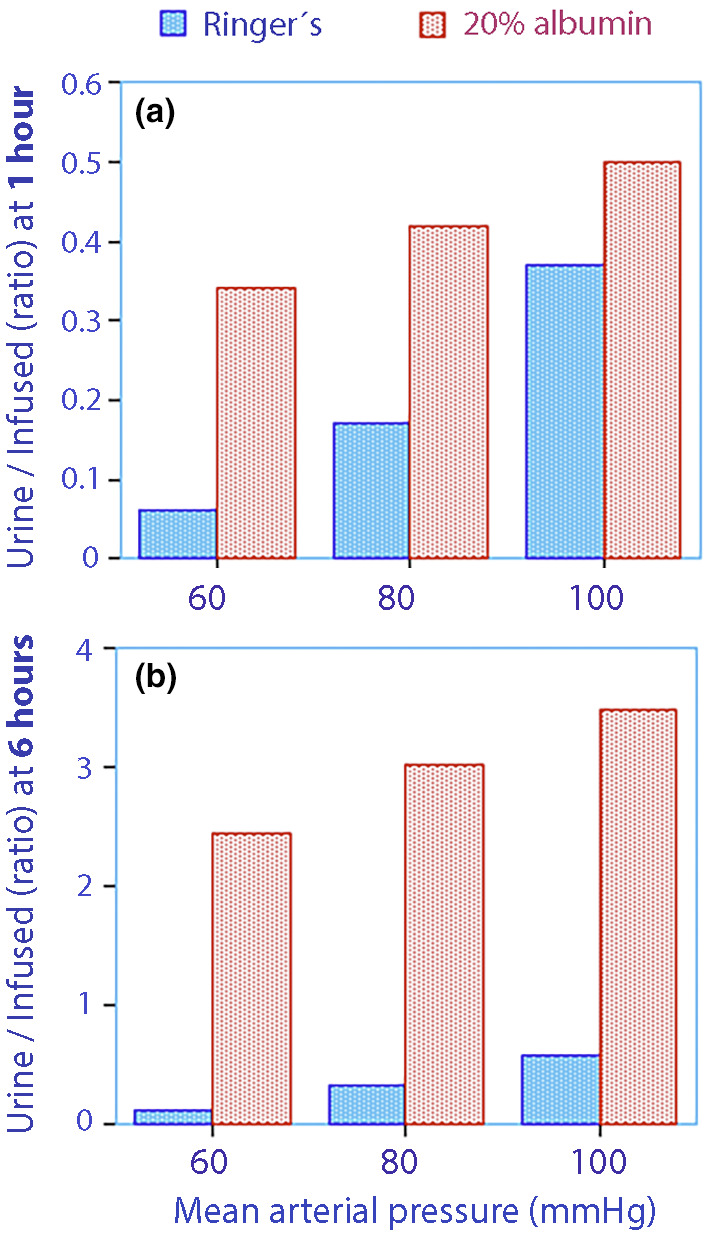
Simulations. (a) The ratio between cumulative urine output and the infused fluid volume for low, normal, and high mean arterial pressures when the plasma volume expansion is maintained at 300 mL over 1 h. The urine output is predicted by multiplication of the y‐axis by the infused fluid volume. (b) Same plot when the plasma volume expansion is maintained for 6 h.

Both fluids created a maximum PV expansion of 15%–25% but their half‐lives differed greatly, which is highlighted by the simulations shown in Figure 5. Here, all kinetic parameters and covariates were used to predict the diuretic responses to Ringer's solution and 20% albumin at three MAP levels (60, 80, and 100 mmHg) when the PV expansion was fixed at 300 mL. These levels were arbitrary but reflect the distribution of the collected data.

The diuretic response was greater for 20% albumin when the MAP was 60 mmHg, while the difference was negligible when MAP was 100 mmHg (Figure 5a).

Albumin was consistently more effective at inducing diuresis when this simulation is extended from 1 to 6 h (compare Figure 5b with Figure 5a). The greater difference between the fluids in this extended simulation is mainly due to that far less volume of 20% albumin than Ringer is needed to maintain a constant PV expansion of 300 mL during 6 h.

The comparisons made in Figure 5 should not be confused with the plots of *k*_10_ shown in Figure 3; the latter relate the urine output in response to any plasma volume expansion regardless of infused volume.

## 4 DISCUSSION

### 4.1 Key findings

The present study contrasts the effectiveness in inducing diuresis between a large‐volume crystalloid fluid (Ringer) with a low‐volume colloid fluid (20% albumin). The results suggest that the PV expansion resulting from infusion of 20% albumin is most effective at inducing diuresis when MAP is low and/or the observation time is long, while Ringer is more effective at a normal or high MAP and the observation time is short (up to 1 h). Hence, 20% albumin might be considered for induction of diuresis in patients who have low MAP and are suspected of being hypovolemic.

Two approaches were used to reach these conclusions. First, the effectiveness of Ringer and 20% albumin at inducing diuresis was studied by examining the correlations between *k*_10_ and MAP (Figure 3) and, second, the importance of time was disclosed by simulations (Figure 5).

### 4.2 Measures of diuretic response

The key variable used to quantify the diuretic response was the volume kinetic parameter *k*_10_, which is the urine flow per minute resulting from a standardized PV expansion (both measured in mL). The logic behind using this parameter is that a greater PV expansion is expected to induce a greater urine output while the PV expansion over time cannot be uncritically extrapolated from an infused fluid volume. Thus, *k*_10_ provides a more physiologically attractive quantification of the diuretic response than measuring urine output alone or after correction for infused volume. This parameter is free from several confounders when assessing diuretic response, as described in the Section 2, and calculated similarly for both fluids. The downside of the approach is that a complete kinetic analysis of how the infused fluid distributes in the body is needed to obtain *k*_10_. On the contrary, the derived set of kinetic parameters opens the possibility of deeper understanding of the data by simulating experiments not performed, as was also done in the present study.

### 4.3 Simulations

Simulations were based on the relationship between the urine output and the infused volume required to maintain a fixed predefined PV expansion. We chose 300 mL to be the target volume expansion because it is the steady state expansion resulting from infusion of 5 mL/kg/h Ringer's solution, which is a common recommendation during surgery (Hahn, 2020).

Again, the effectiveness of 20% albumin in promoting urinary excretion was better when MAP was low (Figure 5a) and even showed overall superiority for lengthy infusions (Figure 5b). The reason for this difference is probably that Ringer's is a less effective PV expander than is 20% albumin.

### 4.4 Predictors of urine flow

The kinetic analysis showed good agreement between the measured and modeled data (Figure 2). This confirms that the choice of models was adequate and that the covariates corrected for the differences between the study groups and the studied subjects, whereby the most precise value of *k*_10_ in each participant could be obtained. The diuretic effects of Ringer's solution were nicely defined by the model (Figure 2b), while the precision was slightly poorer for 20% albumin (Figure 2e).

The covariance analysis showed that excretion of Ringer's was clearly reduced by a low MAP, while general anesthesia *per se* exerted only a minor additional effect. This means that oliguria can mostly be attributed to a low MAP when Ringer's is used as infusion fluid and kidney function is normal (impaired kidney function was a global exclusion criterion).

The diuretic response to 20% albumin was also reduced by a low MAP, but the urinary creatinine concentration, which is biomarker of body hydration, was also a strong predictor (Figure 4b). Hyper‐oncotic albumin increases the urine output in volunteers, which is probably explained by improved renal perfusion (Zdolsek et al., 2018), but the response is thus poorer if the urine is highly concentrated. High levels of creatinine in the urine are often considered to indicate dehydration (Popowski et al., 2001) or low habitual intake of water (Hahn, 2021; Perrier et al., 2013).

### 4.5 Risk of anuria

Development of oliguria/anuria is a paradoxical adverse effect of large amounts of hyper‐oncotic colloids. Rozich & Paul (1989) reported anuria in a cirrhotic patient after administration of 1.8 L of hyper‐oncotic albumin over 72 h. Moran & Kapsner (1987) and Ferraboli et al. (1997) described three patients who became anuric after receiving 100 g of dextran‐40 daily for 1 week. The colloid osmotic pressure during anuria was 33 mmHg (Moran & Kapsner, 1987) (25 mmHg is normal). All three conditions resolved after plasmapheresis. The anuria was probably due to low glomerular filtration rate due to the albumin‐induced increase in colloid osmotic pressure, which otherwise seems to be outweighed by improved renal perfusion.

A more recent population study shows an increased risk of renal events (Schortgen et al., 2008), while other studies do not support this view; the randomized ALBIOS trial of septic patients (Caironi et al., 2014), as well as three literature reviews (Jacob et al., 2008; Jakob, 2004; Wiedermann et al., 2010), report the same incidence of AKI after treatment with 20% albumin compared to crystalloids.

### 4.6 Kinetic analysis

The study used three measured key variables: plasma dilution, MAP, and urine output. The method chosen to connect them was population pharmacokinetics using log‐likelihood mathematics, which is a conventional tool for evaluating drug therapy. However, pharmacokinetics is based on plasma drug concentrations while population volume kinetics uses the measured urinary excretion and changes in Hb concentration to estimate the distribution of infused fluid (Hahn, 2020). A benefit over radio‐isotope tracers is that dynamic events can be studied and simulated over time.

The most important rate parameter in the present study, *k*_10_, was the same in both kinetic models, which allowed them to be compared. Covariates were often measured several times during experiments, which made them serve as “time‐varying covariates” (Owen & Fiedler‐Kelly, 2014). For example, MAP and plasma dilution were always measured at the same points in time. Therefore, a single low MAP provided statistical information for the kinetic analysis even if it was hidden in a higher mean value for all measurements during that experiment. In this way, valid simulations of the diuretic responses to PV expansion at various specific MAPs could be performed if they were within the range of measured values.

Several potential covariates, such as advanced age and sex, did not quite fulfill the required criteria for inclusion in the kinetic models. Other covariates were competitive as they reflected the same physiological event. Plasma albumin could be replaced by the colloid osmotic pressure and urine creatinine by urine osmolality as covariates for *k*_10_ in the albumin model (Figure S2 in File S1). Replacement of urinary creatinine and plasma albumin with the alternatives yielded similar improvements of the kinetic model. The choices we made for this report were based on the more widespread availability of bedside analysis equipment for measurement of these variables.

### 4.7 Limitations

Urinary output is known to be a direct although nonlinear function of arterial pressure with urine flow decreasing to very low levels at pressures below the autoregulatory range, which is about 70 mm Hg. However, the present study shows that the steepness of the curve illustrating the relationship between diuresis and MAP differs greatly depending on the choice of infusion fluid used to stimulate diuresis. This conclusion is based on covariate analyses of an almost equal number of volunteers and surgical patients. The main limitation is that data from these different clinical settings were pooled. However, all experiments planned and managed by the first author and were performed for the same purpose. All data collected in a similar manner, which ensured a low between‐subject and between‐group variability. No subjects with hypovolemia or poor kidney function were included.

Another limitation is that neuro‐humoral mechanisms known to influence urine output that were not assessed. The studied variables were PV expansion, MAP, and the a few other covariates. Other factors are likely to explain much of the variability in Figure 3.

The reported kinetic parameters represent average values throughout an experiment while we cannot rule out that they varied over time.

The rate constants express flow rates induced by the PV expansion resulting from infusion of Ringer's solution and 20% albumin. Baseline rates were not included.

## 5 CONCLUSION

Ringer's solution effectively increases diuresis when MAP values are average (80 mmHg) and higher than average and is suitable for short observation periods (1 h). The urine output per infused volume of 20% albumin is greater when MAP is low (<70 mmHg) and consistently greater when a long‐lasting diuretic effect is desired (6 h). However, highly concentrated urine might attenuate its diuretic effect.

## AUTHOR CONTRIBUTIONS

Robert G Hahn planned the included studies, made the calculations, and authored the manuscript. Markus Zdolsek collected the data during the volunteer experiments with 20% albumin, Michaela Gunnström performed the albumin experiments during surgery, and Emma Hasselgren the experiments during the postoperative period. Joachim H Zdolsek made the applications and assisted with planning and performing the albumin experiments. All authors read and approved the final version of the manuscript.

## FUNDING INFORMATION

Departmental funds were used for this study.

## CONFLICT OF INTEREST STATEMENT

RGH received the Albus Award 2023 grant from Grifols for a study of 20% albumin as infusion fluid during surgery. The other authors declare that they have no conflict of interest.

## ETHICS STATEMENT

All experiments were performed in accordance with the Declaration of Helsinki. The Ethics Committee approvals and database registrations for the 12 studies were the following: Regional Ethics Committee of Huddinge Hospital, Sweden, Nr 54/95, on 1995‐03‐06. Chairman Lennart Kaijser, (Drobin & Hahn, 1999); Regional Ethics Committee of Huddinge Hospital, Sweden, Nr 222/98, on 1998‐06‐02. Chairman Lennart Kaijser, (Drobin & Hahn, 2002); Local Ethics Committee of Karolinska Institutet, Sweden, Nr 97/123, on 1998‐01‐19. Chairman Paul Hjemdahl, (Hahn et al., 2011); Ethics Committee of Linköping University (Ref. M114‐09) on 2009‐06‐17. Chairman Brita Swan. Registered at ClinicalTrials.gov as NCT01062776 on February 4, 2010, (Zdolsek et al., 2012); Regional Ethics Committee of Huddinge Hospital, Sweden, Nr 56/00, on 2000‐02‐07. Chairman Lennart Kaijser, (Svensén et al., 1999); Regional Ethics Committee of Huddinge Hospital, Sweden, Nr 269/02, on 2002‐09‐02. Chairman Hans Glaumann, (Ewaldsson & Hahn, 2005); Local Ethics Committee of Karolinska Institutet, Sweden, Nr 56/00, on 1998‐01‐19. Chairman Lennart Kaijser, (Hahn & Olsson, 2022); Ethics Committee of Zhejiang University (Hangzhou, PR of China, Ref. 080186) on 2008‐01‐10. Chairman Qiu Yongqing, (Li et al., 2012); Regional Ethics Committee in Stockholm, Sweden (Dnr 2014/2146–31/1) on 2015‐04‐28, Chairman Pierre Lafolie, and registered at ClinicalTrials.gov, as identifier NCT02556580 on September 22, 2015, and Eu‐nr 2016–000996‐26, (Hasselgren et al., 2019); Regional Ethics Committee of Linköping (Dnr 2017/478–31) on 2018‐06‐07. Chairman Gunilla Robertsson. Approved by the Swedish Medical Products Agency Eudra‐CT 2017–003687‐12 on September 22, 2017, (Zdolsek et al., 2022); Regional Ethics Committee of Linköping (Dnr 2016/333–32) on 2016‐09‐01. Chairman Staffan Hägg. Registration at Clinicaltrials.gov as NCT02952378 on October 25, 2011, and Eu‐nr 2016–000996‐26 (Li et al., 2012); Regional Ethics Committee in Stockholm, Sweden (Dnr 2014/2146–31/1) on 2015‐04‐28. Chairman Pierre Lafolie. Registered at ClinicalTrials.gov, as identifier NCT02556580, and Eu‐nr 2016–000996‐26; (Zdolsek et al., 2020).

## Supporting information


File S1.

File S2.

File S3.

## Data Availability

The original data are shown in Files S2 and S3.
