# Supplementary material for: Diuretic responses to Ringer's solution and 20% albumin at different arterial pressures
Source: Physiol Rep. 2024 Oct 7;12(19):e70069. doi: 10.14814/phy2.70069 (PMC11458327; doi:10.14814/phy2.70069)
Supplement: Supplementary file 1 — File S1. [file PHY2-12-e70069-s001.docx]

Supplementary File 1

Diuretic responses to Ringer and 20% albumin at different arterial pressures

**
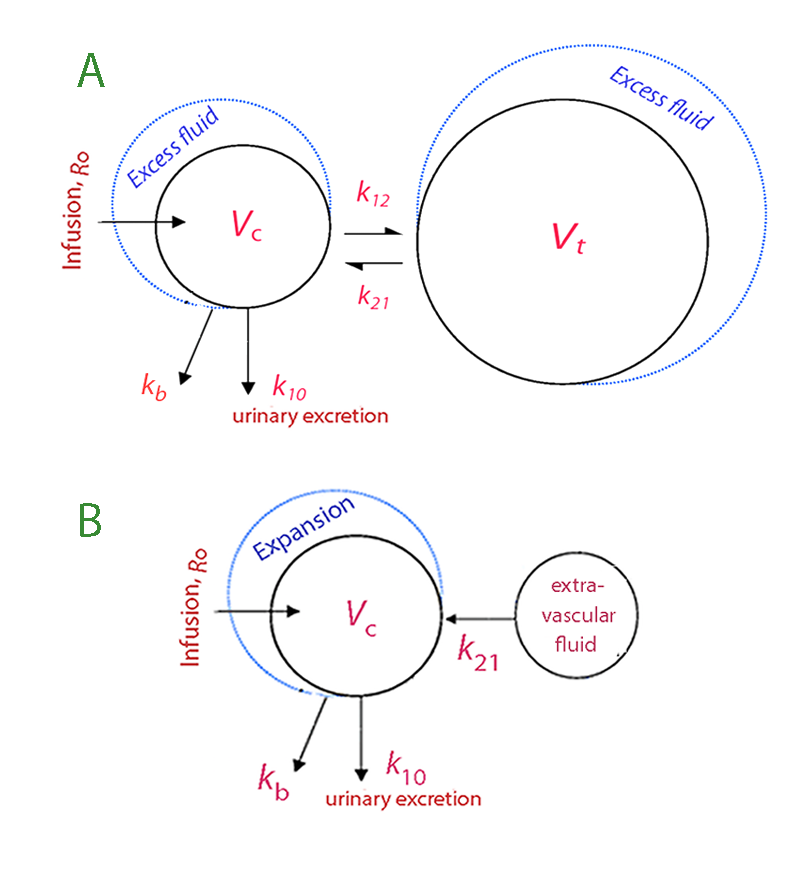
**

**Fig. S1.** **Kinetic models.**

Schematic drawings of the models used to analyze the volume kinetics of

**(A)** crystalloid fluid, and **(B)** 20% albumin.

**Model equations for crystalloid fluid**

*The description relates to Fig. S1A above.*

The volume kinetic model implies that fluid is infused at the rate *R*_o_ into the plasma (*V*_c_) and that the fluid is then distributed to (*k*_12_) and re-distributed from (*k*_21_) to the extravascular space (*V*_t_). Elimination occurs from *V*_c_ by urinary excretion (*k*_10_) or by deposition in the "third space" (*k*_b_). All flow rates are proportional, by one of these rate constants (*k*_12_, *k*_21_, *k*_10,_ and *k*_b_), to the volume expansion of the respective body fluid space. No lag times were modeled. The differential equations for the kinetic model are:

 d*v*_c_ /dt = *R*_o_ – *k*_12_ (*v*_c_ – *V*_c_) + *k*_21_ (*v*_t_ – *V*_t_) – *k*_10_ (*v*_c_ – *V*_c_) – *k*_b_ (*v*_c_ – *V*_c_)

d*v*_t_ /dt = *k*_12_ (*v*_c_ – *V*_c_) – *k*_21_ (*v*_t_ – *V*_t_)

dU /dt = *k*_10_ (*v*_c_ – *V*_c_)

Baseline volumes are given in capital letters (*V*_c_ and *V*_t_), expanded volumes in lower-case letters (*v*_c_ and *v*_t_), and U is the urinary excretion. Hence, volume expansion of the central fluid space is given by (*v*_c_ – *V*_c_). The glycocalyx water is part of *V*_c_.

The Hgb-derived fractional plasma dilution used to indicate the volume expansion of *V*_c_ resulting from the infusion:

(*v*_c_ – *V*_c_) / *V*_c_ = [(Hgb / hgb) – 1)] / (1 – baseline hematocrit)

The slow re-distribution of fluid from the deep compartment (“third space”) is embedded in *k*_21_, although a separate quantification of this flow using two rate constants is technically possible (see *Microvascular Research* 2023: 151: 104599). However, practically the same *k*_10_ was obtained when fitting the data to a model where two rate constants were used (the analysis can be sent on reasonable request to Robert.hahn@ki.se).

**Model equations for 20% albumin**

*The description relates to Fig. S1B above.*

Fluid is infused by a rate *R*_o_ into the central body fluid space *V*_c_, which is then expanded to *v*_c_. Fluid is recruited from the interstitial fluid space volume (IFV; assumed to be 15% of the body weight; see Guyton AC, Hall JE: *Textbook of Medical Physiology.* 9th edition. Philadelphia: WB Saunders Co, 1996, pp. 297–313) to the plasma, which is due to the increase in oncotic pressure resulting from the infused excess of albumin molecules. The rate of the absorption is governed by a rate constant *k*_21_.

Elimination of fluid occurs by urinary excretion and capillary leakage at rates proportional to the expansion of *V*_c_ by the rate constants *k*_10_ and *k*_b_. The finally developed ‘base model’ was expressed by the following equations, where *u* denotes the measured urinary excretion (the term *k*_21_ IFV applies only when the infusion has started):

d*v*_c_ /dt = *R*_o_ – *k*_b_ (*v*_c_ – *V*_c_) – *k*_10_ (*v*_c_ – *V*_c_) + *k*_21_ IFV

dIFV/dt = – *k*_21_ IFV_o_

d*u*/dt = *k*_10_ (*v*_c_ – *V*_c_)

The rate constant *k*_21_ is only operational when 20% albumin is or has been infused. This model has been described and validated in: Hahn RG, Zdolsek M, Hasselgren E, Zdolsek J, Björne H. *Br J Clin Pharmacol* 2019; 85: 1303–1311.

Note that the albumin model does not include return flow from the interstitium to the plasma (*k*_21_) that is driven by the volume expansion of the extravascular space, which is the case in the Ringer model. Here, the return flow is driven by the rise of the plasma oncotic pressure resulting from the hyper-oncotic composition of the fluid. It is probable that fluid leaking to the extravascular space by *k*_b_ returns to *V*_c_ via *k*_21_ but current kinetic analyses are not able to confirm this view.

**Plasma dilution**

The Hgb-derived fractional plasma dilution used to indicate the volume expansion of *V*_c_ resulting from the infusion. The basic equation for this relationship is:

(*v*_c_ – *V*_c_) / *V*_c_ = [(Hgb / Hgb) – 1)] / (1 – baseline hematocrit)

Derivation of the basic equation expressing plasma dilution is given in Supplementary Data File 1 appended to *Anesth Analg* 2022; **134:**1270–1279.

This calculation of plasma dilution should be corrected for the losses of Hgb with blood sampling and surgical hemorrhage. This was done by estimating the total mass of hemoglobin (MHgb), which is the product of the blood Hgb concentration (Hgb) and the total blood volume (BV), and subtracting the losses of Hgb, which are given by the product of the lost blood volume and the average Hgb measured during the same period [Hahn RG: *Acta Anaesthesiol Scand* 1987; **31:**572–578]. The BV at baseline (Time 0) was estimated by Nadler’s anthropometric formula. Hence, between Time 0 and a later Time t:

MHgb_o_ = BV_o_ Hgb_o._

MHgb_t_ = MHgb_o_ – Hgb_loss._ ****

BV_t_ = MHgb_t_ / Hgb_t_ ****

The plasma volume at baseline (PV_o_) was given by BV_o_ (1 – hematocrit).

The initial blood volume was obtained by using Nadler’s formula, where the total blood volume prior to the infusion of 20% albumin (BV_0_) was derived from the height (*h*) in metres, weight (*w*) in kilograms and sex with the following equations [Nadler, Hidalgo & Bloch: Surgery 1962;51:224-232]:

| Male: $\mathrm{BV}_{o} = 0.3669 h^{3} + 0.03219 w + 0.6041$ |
| --- |
| Female: $\mathrm{BV}_{o}= 0.3561 h^{3} + 0.03308 w + 0.1833$ |

Sensitivity analysis has shown that an erroneous estimate of BV_o_ to 80% and 120% of the correct value has only a small effect on the numerical correction of plasma dilution for Hgb losses [*Anesthesiology* 1999; **90:**81–91

**Covariate analysis**

The fluid kinetics for each group of subjects is given by parameters in the base models: *V*_c_, *k*_12_, *k*_21_*, k*_10_, and *k*_b_ for Ringer’s solution and *V*_c_, *k*_10_, *k*_b_*,* and *k*_21_ for 20% albumin (see Fig. 1 of the manuscript). The parameter value for an individual subject could be modified by inclusion of *covariates*. Appropriate candidates for inclusion as covariates were sought using plots of random effects ("eta:s") and validated by adding them one by one to the model according to the exponential covariate model for dichotomized variables or the power covariate model for continuous variables. The evaluated candidates for covariance in the Ringer’s infusions were Hb at baseline, body weight, body mass index (BMI), age, sex, MAP, and ongoing general anesthesia. The candidates for 20% albumin were the same as for the Ringer’s experiments but also included the change in plasma albumin from baseline, plasma creatinine, plasma colloid osmotic pressure, and urine creatinine and urine osmolality at baseline.

The principles for this analytical approach are explained in detail in: Owen JS, Fiedler-Kelly J. *Introduction to population pharmacokinetic/pharmacodynamic analysis with nonlinear mixed effects models.* Hoboken: Wiley & Sons, 2014.

Testing was made in sequence to all parameters in the base model. The covariate was accepted for inclusion if it significantly improved the goodness-of-fit for the model; a reduction of -2 LL (LL = log likelihood) by >3.8 points represents *P*< 0.05 and >6.6 points represents *P*< 0.01. Furthermore, zero was not allowed to be within the 95% confidence interval of the covariate, and the between-subject variability had to be < 50%. The fixed parameters in the base model and the statistically significant covariates were always estimated simultaneously using the Phoenix software.

The ***exponential covariate model*** was used to analyze the influence of dichotomized variables on the parameters in the base model. For example, the rate parameter *k*_10_ has the group value of 0.0137 min^-1^ for crystalloid fluid, but the value is different during general anesthesia with a computer-generated covariate effect of -1.13. The equation for *k*_10_ for an individual (ind) then becomes:

*k*_10_ _ind_ = *k*_10_ _group_ [e ^awake=0, anesthesia= -1.13^]

where e= 2.718. In an awake subject, *k*_10_ collapses to the group value of 0.0137 min^-1^ because (e^0 = 1). By contrast, *k*_10_ becomes 0.0137*(2.718^ -1.13) = 0.0044, which is only 32% of the value obtained in a conscious subject.

Continuous variables were evaluated according to the ***power model***. For example, the mean body weight was 72.9 kg, the group value for *V*_c_ was 3.82 L, and the covariate effect is 1.70. The individual value of *V*_c_ for a subject weighing 55 kg then becomes:

*V*_c_ _ind_ = 3.82 [(55 / 72.9) ^1.70^] = 2.37 L

The criterion for accepting a covariate was that its inclusion should reduce the -2 LL (log likelihood) for the model by > 6.6 points (*P*< 0.01). In addition, the 95% confidence interval (CI) for the estimate of the covariate was not allowed to include 0.

The data on the Ringer solutions were not sufficiently rich to analyze the association between concentrated urine and *k*_10_. However, a previous study in elderly men (mean age 72 years) confirmed that concentrated urine also attenuates the diuretic response to crystalloid [*Clin Exp Pharm Physiol* 2021; **48:**310–317]. There was also a decrease in *k*_10_ for higher plasma creatinine, which was not possible to analyze here because impaired kidney function was an exclusion criterion.

The return flow from the extravascular space to the plasma is not possible to quantify in the albumin model because the capillary leakage is driven by the PV expansion while the absorption to the PV is driven by increased plasma oncotic press

**Table S1**

**Crystalloid fluid volume kinetics.**

Group values (tv = typical value) for the fixed parameters in the group, followed by individual-specific covariates.

| Kinetic parameter | Covariate | Covariate model | Best estimate | 95% CI | CV% | -2 LL |
| --- | --- | --- | --- | --- | --- | --- |
|  |  |  |  |  |  |  |
| ­­tv*V*_c_ (L) |  |  | 3.82 | 3.49–4.16 | 4.5 |  |
| tv*k*_12_ (10^-3^ min^-1^) |  |  | 51.2 | 39.1–63.5 | 12.1 |  |
| tv*k*_21_ (10^-3^ min^-1^) |  |  | 38.8 | 28.9–48.6 | 13.0 |  |
| tv*k*_10_ (10^-3^ min^-1^) |  |  | 13.7 | 14.5–18.9 | 8.1 | -7009 |
| tv*k*_b_ (10^-3^ min^-1^) |  |  | 7.8 | 5.5–10.0 | 14.9 | -7178 |
| *V*_c_ | Body weight | Power | 1.70 | 1.37–2.04 | 10.1 | -7286 |
| *k*_10_ | MAP | Power | 2.94 | 2.01–3.87 | 16.2 | -7387 |
| *k*_12_ | Hb at baseline | Power | -3.25 | -4.38 to -2.12 | -17.7 | -7397 |
| *k*_10_ | Anesthesia | Exponential | -1.13 | -1.46 to -0.79 | -15,1 | -7452 |
| *V*_c_ | Anesthesia | Exponential | -0.43 | -0.57 to -0.30 | -15.8 | -7481 |
| *k*_21_ | Hb at baseline | Power | -2.60 | -3.73 to -1.46 | -22.3 | -7495­ |
|  |  |  |  |  |  |  |

The mean values of the body weight, MAP, and Hb level are shown in Table 1.

CI = confidence interval. CV% = coefficient of variation (inter-individual). MAP = mean arterial pressure.

LL = log likelihood of the model during development. Decrease in -2 LL by >6.6 points = *P*< 0

**Table S2**

**Fluid volume kinetics of 20% albumin.**

Group values (tv = typical values) for the fixed parameters in the group, followed by individual-specific covariates.

| Kinetic parameter | Covariate | Covariate model | Best estimate | 95% CI | CV% | -2 LL |
| --- | --- | --- | --- | --- | --- | --- |
|  |  |  |  |  |  |  |
| ­­tv*V*_c_ (L) |  |  | 4.25 | 3.22–5.27 | 12.3 |  |
| tv*k*_b_ (10^-3^ min^-1^) |  |  | 26.5 | 23.3–29.7 | 6.2 |  |
| tv*k*_21_ (10^-3^ min^-1^) |  |  | 1.58 | 1.11–20.5 | 15.3 |  |
| tv*k*_10_ (10^-3^ min^-1^) |  |  | 3.98 | 3.06–4.90 | 11.8 | -1697 |
| *k*_10_ | Urine creatinine^1^ | Power | -0.46 | -0.66 to -0.26 | -22.3 | -1720 |
| *k*_10_ | Plasma albumin^2^ | Power | 1.03 | 0.27–1.79 | 37.8 | -1738 |
| *k*_10_ | MAP^3^ | Power | 0.91 | 0.20–1.62 | 39.7 | -1746 |

^1^ Urine creatinine at baseline, mean 12.7 mmol/L (N=84); ^2^ Plasma albumin 3.93 g/dL (N=1,257), ^3^ MAP 81.6 mmHg (N=456).

Urine creatinine could be replaced by urine osmolality in this analysis, see **Fig. S1**.

CI = confidence interval. CV% = coefficient of variation (inter-individual). MAP = mean arterial pressure.

LL = log likelihood of the model during development. Decrease in -2 LL by >6.6 points = *P*< 0.01.

**
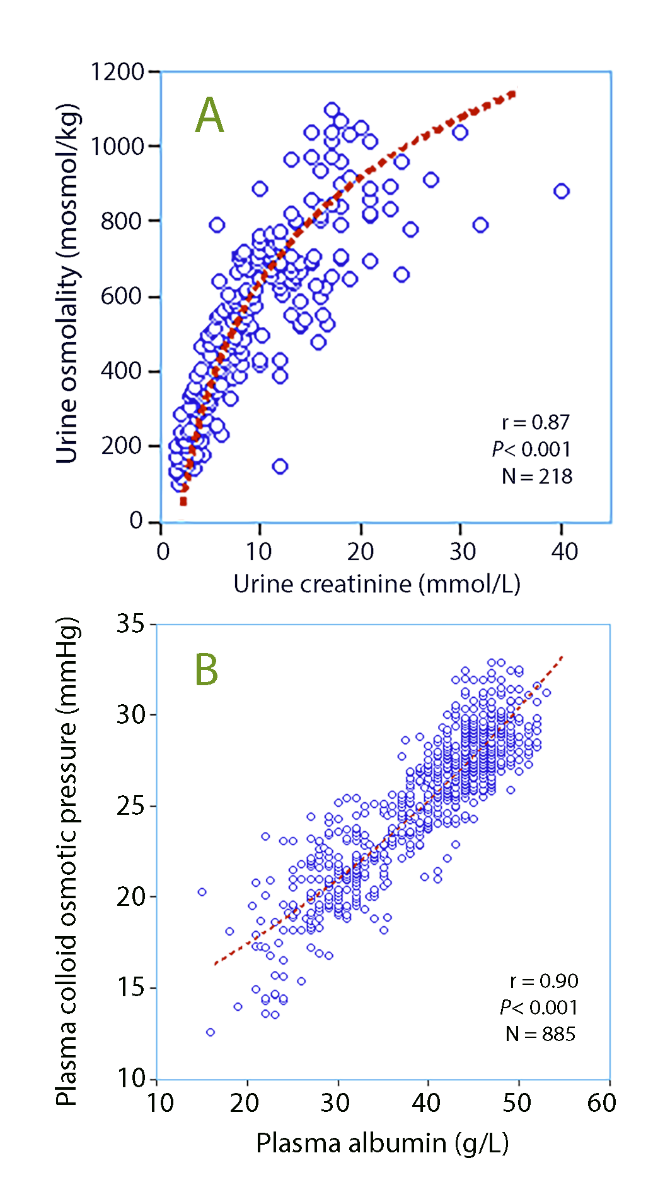
**

**Fig. S2.** **Correlations between competing covariates**

**(A)** Urine creatinine concentration vs*.* urine osmolality. These were measured 3 times during each infusion of 20% albumin, but only the baseline was used in the kinetic analysis. **(B)** Plasma albumin vs. the plasma colloid osmotic pressure.

The data were collected during the 85 experiments with 20% albumin.

Irregular red lines were created by linear regression based on power-transformed and exponential transformation of the data on the y-axis.

MODEL TEXT USED TO ANALYZE THE RINGER KINETICS

deriv(A1 = - (A1 * Ke)- (A1 * K12- A2 * K21)- (A1 * kb))

urinecpt(A0 = (A1 * Ke))

deriv(A2 = (A1 * K12- A2 * K21))

urinecpt(A3 = (A1 * kb))

C = A1 / V

dosepoint(A1, idosevar = A1Dose, infdosevar = A1InfDose, infratevar = A1InfRate)

error(CEps = 0.0261405033642024)

observe(CObs = C + CEps)

error(A0Eps = 87.3869126063085)

observe(A0Obs = A0 + A0Eps)

stparm(V = tvV * (Bodyweight/mean(Bodyweight))^dVdBodyweight * exp(dVdVaken0anest11*(Vaken0anest1==1)) * exp(nV))

stparm(Ke = tvKe * (MAP/mean(MAP))^dKedMAP * exp(dKedVaken0anest11*(Vaken0anest1==1)) * exp(nKe))

stparm(K12 = tvK12 * (Hbo/mean(Hbo))^dK12dHbo * exp(nK12))

stparm(K21 = tvK21 * exp(nK21))

stparm(kb = tvkb * exp(nkb))

fcovariate(Bodyweight)

fcovariate(MAP)

fcovariate(Lapscopi())

fcovariate(GenderMale0())

fcovariate(Hbo)

fcovariate(Age)

fcovariate(Vaken0anest1())

fixef(tvV = c(, 3823.8237379837, ))

fixef(tvKe = c(, 0.0137377025630489, ))

fixef(tvK12 = c(, 0.0512753431552566, ))

fixef(tvK21 = c(, 0.0387684501094251, ))

fixef(tvkb = c(, 0.00776446086914414, ))

fixef(dVdBodyweight(enable=c(0)) = c(, 1.70422976304761, ))

fixef(dKedMAP(enable=c(1)) = c(, 2.93660730707422, ))

fixef(dKedVaken0anest11(enable=c(2)) = c(, -1.13, ))

fixef(dVdVaken0anest11(enable=c(3)) = c(, -0.43, ))

fixef(dK12dHbo(enable=c(4)) = c(, -2.6, ))

ranef(diag(nV, nKe, nK12, nK21, nkb) = c(0.084565025, 0.49727496, 0.30467147, 0.23859748, 0.99885295))

MODEL TEXT USED TO ANALYZE THE KINETICS OF 20% ALBUMIN

deriv(A1 = - (A1 * Ke) + (Aa * kabs)- (A1 * kb))

urinecpt(A0 = (A1 * Ke))

deriv(Aa = - (Aa * kabs))

urinecpt(A2 = (A1 * kb))

C = A1 / V

dosepoint(A1, idosevar = A1Dose, infdosevar = A1InfDose, infratevar = A1InfRate)

error(CEps = 0.0283597437653591)

observe(CObs = C + CEps)

error(A0Eps = 264.600460824285)

observe(A0Obs = A0 + A0Eps)

dosepoint(Aa, idosevar = AaDose, infdosevar = AaInfDose, infratevar = AaInfRate)

stparm(V = tvV * exp(nV))

stparm(Ke = tvKe * (Ucrea/mean(Ucrea))^dKedUcrea * (Albumin/mean(Albumin))^dKedAlbumin * (MAP/mean(MAP))^dKedMAP * exp(nKe))

stparm(kabs = tvkabs * exp(nkabs))

stparm(kb = tvkb * exp(nkb))

fcovariate(Age)

fcovariate(GenderMale0())

fcovariate(Pkreatinin)

fcovariate(BMI)

fcovariate(Albumin0)

fcovariate(Uosm)

fcovariate(Ucrea)

fcovariate(COPstart)

fcovariate(MAP)

fcovariate(COP)

fcovariate(Bodyweight)

fcovariate(Albdiff)

fcovariate(Albumin)

fixef(tvV = c(, 4246.28395045749, ))

fixef(tvKe = c(, 0.00397829793388295, ))

fixef(tvkabs = c(, 0.00157824937073324, ))

fixef(tvkb = c(, 0.0264778353251179, ))

fixef(dKedUcrea(enable=c(0)) = c(, -0.460543012109636, ))

fixef(dKedAlbumin(enable=c(1)) = c(, 1.02902680856117, ))

fixef(dKedMAP(enable=c(2)) = c(, 0.912723855667709, ))

ranef(diag(nV, nkb, nkabs, nKe) = c(0.18966437, 0.099281583, 2.8539435E-07, 4.6642306E-06))
